# IL-1/TNF-*α* Inflammatory and Anti-Inflammatory Synchronization Affects Gingival Stem/Progenitor Cells' Regenerative Attributes

**DOI:** 10.1155/2017/1349481

**Published:** 2017-11-09

**Authors:** Fan Zhang, Misi Si, Huiming Wang, Mohamed K. Mekhemar, Christof E. Dörfer, Karim M. Fawzy El-Sayed

**Affiliations:** ^1^Clinic for Conservative Dentistry and Periodontology, School of Dental Medicine, Christian-Albrechts-Universität zu Kiel, Kiel, Germany; ^2^Stomatology Hospital Affiliated to School of Medicine, Zhejiang University, Zhejiang, China; ^3^Oral Medicine and Periodontology Department, Faculty of Oral and Dental Medicine, Cairo University, Cairo, Egypt

## Abstract

Cytokines play major roles in tissue destruction/repair. The present study investigates proliferative and osteogenic differentiation potentials of gingival mesenchymal stem/progenitor cells (G-MSCs), influenced by IL-1/TNF-*α* inflammatory/anti-inflammatory conditions. Human G-MSCs were isolated, characterized, and cultured in basic medium (control group, M1), in basic medium with IL-1*β*, TNF-*α*, and IFN-*γ* (inflammatory group, M2) and with IL-1ra/TNF-*α*i added to M2 (anti-inflammatory group, M3). MTT tests at days 1, 3, and 7 and CFU assay at day 12 were conducted. Osteogenic differentiation was analyzed by bone-specific transcription factors (RUNX2), alkaline phosphatase (ALP), type I collagen (Col-I), osteopontin (OPN), and osteonectin (ON) expression at days 1, 3, 7, and 14 and Alizarin red staining at day 14. At day 3, the control group showed the highest cell numbers. At day 7, cell numbers in inflammatory and anti-inflammatory group outnumbered the control group. At day 12, CFUs decreased in the inflammatory and anti-inflammatory groups, with altered cellular morphology. The anti-inflammatory group demonstrated elevated bone-specific transcription factors at 14 days. After 14 days of osteogenic induction, calcified nodules in the anti-inflammatory group were higher compared to control and inflammatory groups. For regeneration, initial inflammatory stimuli appear essential for G-MSCs' proliferation. With inflammatory persistence, this positive effect perishes and is followed by a short-term stimulatory one on osteogenesis. At this stage, selective anti-inflammatory intervention could boost G-MSCs' differentiation.

## 1. Introduction

Periodontitis, a highly prevalent inflammatory disease, is characterized by damage to the cementum, alveolar bone, and periodontal ligament [1]. Although its pathological process is initiated by the expression of virulence factors in the oral microbial biofilm, its progression is primarily governed by the host's immune response, with a massive production of inflammatory cytokines, including tumor necrosis factor alpha (TNF-*α*), interleukin-1 beta (IL-1*β*), interleukin-4 (IL-4), interleukin-6 (IL-6), and interferon gamma (IFN-*γ*) [2]. TNF-*α* and IL-1*β*, two of the most important and earliest cytokines appearing during periodontal inflammation, are thereby primarily responsible for the resultant bone resorption and periodontal destructive process [3]. Interferon gamma (IFN-*γ*), whose gingival cervical fluid level is considered to be related to the severity of periodontal disease, is a further signature inflammatory cytokine appearing during the periodontal destructive process [4]. These inflammatory biomolecules could regulate the periodontal cells' survival, behaviour, and activity, and thereby the duration, extent, and outcome of the disease or its therapy [5]. Anti-inflammatory cytokines conversely participate in dictating the clinical course of periodontal pathological conditions. As a naturally secreted antagonist to the actions of IL-1, IL-1 receptor antagonist (IL-1ra) has been found to play a protective role in experimental periodontitis [6]. In addition, TNF-*α* inhibitor (TNF-*α*i), a chemosynthetic competitive antibody of TNF-*α*, could inhibit inflammatory bone resorption, when systemically administrated in an animal model of experimental periodontitis [7]. However, the combined therapeutic effect of these two pivotal anti-inflammatory cytokines on cellular attributes, in an *in vitro* inflammatory environment-mimicking periodontitis, has not been investigated, yet.

Human gingival stem/progenitor cells (G-MSCs) show a great periodontal regenerative potential and hold hope for future clinical applications [8–10]. During their transplantation into the periodontal tissue, G-MSCs' attributes and behaviour are influenced by their surrounding inflammatory/anti-inflammatory microenvironment [11]. Currently, controversies exist regarding the impact of inflammation and the virtue of its suppression on the tissue regenerative potential of G-MSCs [10, 12, 13]. The present study aimed to evaluate for the first time the effect of the anti-inflammatory cytokines IL-1ra and TNF-*α*i, on the proliferation and the osteogenic potential of G-MSCs cultured under inflammatory conditions *in vitro*.

## 2. Materials and Methods

### 2.1. G-MSC Isolation

G-MSCs were obtained from the healthy gingival collars around partially impacted third molars at the Christian-Albrechts University of Kiel, Germany. The study was performed in accordance with Helsinki Declaration revised in 2008 and approved by the Ethics Committee of Christian-Albrechts University of Kiel, Germany. Cells' isolation and culture were done as formerly described [14, 15]. Briefly, gingival soft tissue collars were detached, deepithelized, cut into pieces, and washed in a *basic medium*, consisting of Eagle's minimum essential medium alpha modification (Sigma-Aldrich GmbH, Hamburg, Germany), with 15% fetal calf serum (FCS, HyClone, Logan, UT, USA), 400 mmol/ml L*-*glutamine, 100 U/ml penicillin, 100 *μ*g/ml streptomycin, and 1% amphotericin (all from Biochrom, Berlin, Germany). The soft tissue parts were left to adhere for 30 min in dry tilted culture flasks, before the *basic medium* was slowly added to them. The flasks were incubated at 37°C with 5% CO_2_ to let the cells grow and reach 80% confluence. The *basic medium* was replaced three times/week.

Immunomagnetic cell sorting employing anti-STRO-1 antibody (BioLegend, San Diego, CA, USA) and anti-IgM MicroBeads (Miltenyi Biotec, Bergisch Gladbach, Germany) were used to isolate the G-MSCs as described before [14]. The obtained G-MSCs were cultured in a *basic medium*.

### 2.2. Flow Cytometric Analysis (FACS)

The second passage G-MSCs were incubated with the primary antibodies for CD14, CD34, CD45, CD73, CD90, CD105, CD146 (Becton Dickinson, Heidelberg, Germany), and the corresponding isotypes according to the manufacturers' instruction. FACSCalibur E6370 and FACSComp 5.1.1 software (Becton Dickinson) were used to evaluate the expressions.

### 2.3. Multilineage Differentiation

- Osteogenic differentiation: the econd passage G-MSCs were seeded in six-well culture plates at a density of 2 × 10^4^ cells/well. After cell adhesion, osteogenic inductive medium (PromoCell, Heidelberg, Germany) was added and changed three times/week. G-MSCs cultured at the same density in a *basic medium* were used as controls. After 14 days, Alizarin red (Sigma-Aldrich) staining of both groups was used to assess extracellular matrix calcification.
- Adipogenic differentiation: the second passage G-MSCs were seeded into six-well culture plates at a concentration of 3 × 10^5^ cells/well. Following cell adhesion, adipogenic inductive medium (PromoCell) was added and changed three times/week. G-MSCs cultured at the same density in a *basic medium* were used as controls. Oil Red O staining (Sigma-Aldrich) was used to evaluate the presence of lipid drops in both groups after 21 days.
- Chondrogenic differentiation: the second passage G-MSCs were cultured in 1.5 ml Eppendorf tubes (Eppendorf, Hamburg, Germany) as 3D micromasses at a density of 3 × 10^4^cells/tube in chondrogenic inductive medium (PromoCell). G-MSCs cultured at the same density in a *basic medium* were used as controls. The medium was changed three times/week. After 35 days, Alcian blue and nuclear-fast-red counterstaining (Sigma-Aldrich) of the sectioned 3D micromasses of both groups was used to evaluate the formation of glycosaminoglycans.

### 2.4. Experimental Groups

The second passage G-MSCs were cultured in three different media. The media were renewed three times/week:

- Control group (M1): *basic medium*
- Inflammatory group (M2): inflammatory medium consisting of a *basic medium*, supplemented with the key proinflammatory cytokines observed during periodontitis: IL-1*β* (1 ng/ml), TNF-*α* (10 ng/ml), and IFN-*γ* (100 ng/ml) (PeproTech Inc, Rocky Hill, NJ, USA) [16]
- Anti-inflammatory group (M3): inflammatory medium supplemented with the anti-inflammatory cytokines: IL-1ra (10 ng/ml) (R&D systems, Minneapolis, MN, USA) and TNF-*α*i (10 ng/ml) (Enzo Life Sciences GmbH, Lörrach, Germany)

### 2.5. Colony-Forming Unit (CFU) Assay

The second passage G-MSCs were seeded in 10 cm diameter culture dishes at a density of 1.5 × 10^3^ cells/dish according to the group allocation outlined above. On day 12, cell cultures were fixed with ice-cold 100% methanol for 10 min and stained with 0.1% crystal violet for 10 min. CFUs were assessed by two different observers under the phase contrast inverted microscopy. Aggregations of 50 or more cells were scored as one colony.

### 2.6. Cell Proliferation Assays

Cell proliferation and viability were determined using the MTT (3-[4,5-dimethylthiazol-2-yl]-2,5-diphenyl-tetrazoliumbromide) cell proliferation kit-I (Roche Diagnostics GmbH, Roche Applied Science, Mannheim, Germany) as described before [17]. The second passage G-MSCs were seeded in 24-well culture plates at a density of 1 × 10^4^ cell/well. After adhesion (day 0), cells were cultured according to the group allocation described above. On days 1, 3, and 7, the media were replaced by no phenol red serum-free medium (RPMI 1640, PAN-Biotech GmbH, Aidenbach, Germany). 100 *μ*l of the MTT labelling reagent (final concentration 0.5 mg/ml) was added into each well and incubated for 4 hrs (37°C, 5% CO_2_). 1 ml of the solubilization solution was added into each well and incubated overnight (37°C, 5% CO_2_). The spectrophotometrical absorbance of the samples in each well was measured, using a universal microplate spectrophotometer (*μ*Quant, BioTek Instruments GmbH, Vermont, USA) at 570 nm wavelength. Relevant cell numbers were calculated by comparing with the standard curve. Each assay was performed in triplicate and averaged.

### 2.7. Osteogenic Differentiation

To investigate the effect of proinflammatory and anti-inflammatory cytokines on G-MSCs' osteogenic potential, the second passage G-MSCs were seeded in 6-well plates at a density of 2 × 10^4^ cells/well in a *basic medium*. After cell adhesion (day 0), the media were replaced with osteogenic inductive medium according to group allocation described above for 14 days. On day 14, *in vitro* mineralization was assessed by Alizarin red staining of representative cultures.

On days 1, 3, 7, and 14, the total RNA of cell culture was extracted by RNeasy kit (Qiagen, Hilden, Germany) and purified by QuantiTect Reverse Transcription Kit (Qiagen). Reverse transcription was used to synthesize complementary cDNA by QuantiTect Reverse Transcription Kit (Qiagen). The expression of the bone-specific transcription factor RUNX2, alkaline phosphatase (ALP), type I collagen (Col-I), and osteopontin (OPN) was measured using real-time quantitative polymerase chain reaction (real-time PCR or qPCR) by RealTime ready assays (Roche LightCycle® 96, Roche Molecular Biochemicals, Indianapolis, IN, USA). The house-keeping gene GAPDH was used as a reference to calculate relative gene expression. The designed primers were supplied by Roche (Table 1). All experiments were performed in triplicate and averaged.

### 2.8. Statistical Analysis

Data was analyzed using the SPSS 20.0 software (IBM Corporation, NY, USA). Median and interquartile range was calculated for quantitative variables. Differences in cell numbers and gene expression among the three groups (*n* = 3/group) were examined using nonparametric analysis methods since the data were nonnormally distributed. The homogeneity of variances was verified. The Kruskal-Wallis test was performed to analyze the data with homogeneity across groups. Mood's median test was applied for data without homogeneity. Dunn's test was performed as a post hoc multiple comparisons. The significance level was set at 0.05.

## 3. Results

### 3.1. Cell Culture and Identification

**Figure 1:**
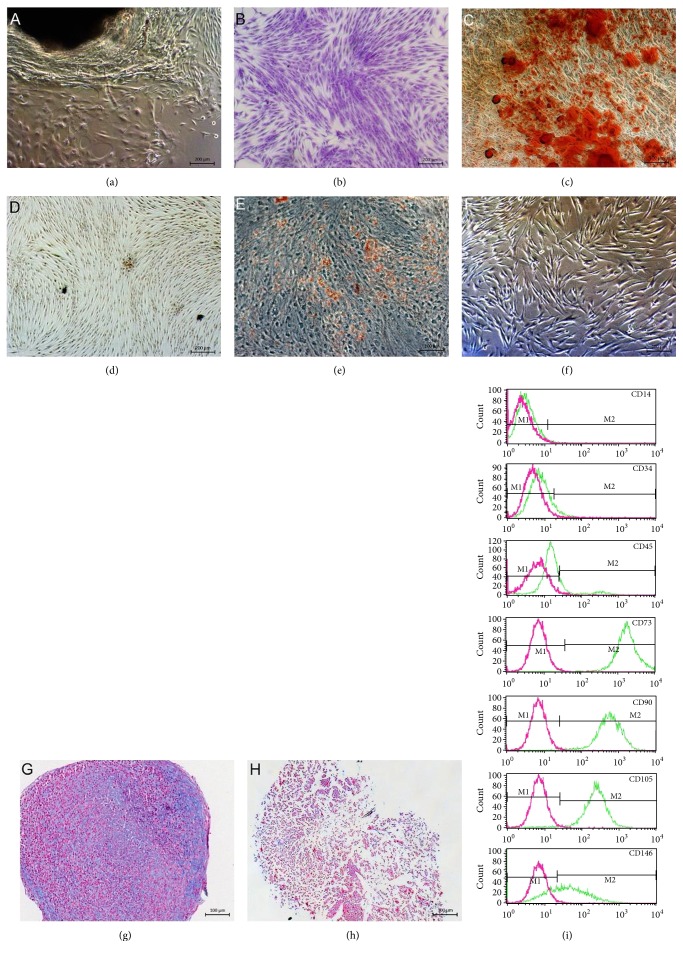
Isolation and characterization of G-MSCs. (a) Microscopic appearance of outgrowing cells from free gingival margin. (b) Microscopic appearance of G-MSCs stained with crystal violet. (c) Alizarin red staining of G-MSCs after osteogenic induction. (d) Alizarin red staining of G-MSCs cultured in a *basic medium*. (e) Oil Red O staining of G-MSCs after adipogenic stimulation. (f) Oil Red O staining of G-MSCs cultured in a *basic medium*. (g) Alcian blue staining of G-MSCS after chondrogenic stimulation. (h) Alcian blue staining of G-MSCs cultured in a *basic medium*. (i) Flow cytometry analysis of cell surface markers of G-MSCs.

Following their outgrowth (Figure 1(a)), human G-MSC cultures presented adherent fibroblast-like clusters (Figure 1(b)) and multilineage differentiation capacity into osteogenic (Figures 1(c) and 1(d)), adipogenic (Figures 1(e) and 1(f)), and chondrogenic (Figures 1(g) and 1(h)) directions. G-MSCs showed a surface marker expression profile characteristic for MSCs, including a positive expression of the mesenchymal stem/progenitor cells' surface markers CD73, CD90, CD105, and CD146 and a negative expression of CD14, CD34, and CD45 (Figure 1(i)).

### 3.2. G-MSC Proliferation and Colony Formation under Inflammatory/Anti-Inflammatory Environment

**Figure 2:**
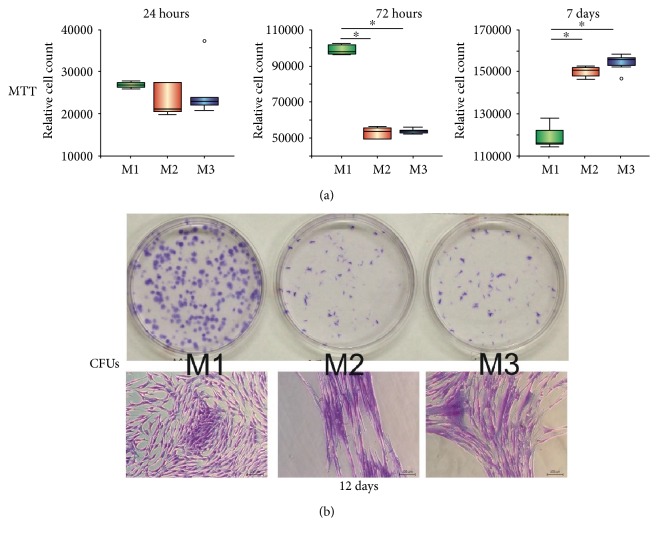
Cell proliferation and colony-forming characteristics of G-MSCs under inflammatory environment. (a) MTT results of G-MSC proliferation in the three groups at day 1, 3, and 7 days. Significant differences between groups are marked with an asterisk (^∗^*p* < 0.05; Dunn's test). (b) Crystal violet staining results for colony-forming unit (CFU) assay and microscopic appearance of G-MSCs cultured with a basic medium (M1), inflammatory medium (M2), and anti-inflammatory treatment medium (M3) after 12 days.

MTT results showed that cell numbers in all three groups were similar at day 1 (*p* = 0.472). Cell numbers were significantly lower in the inflammatory group (M2) and anti-inflammatory group (M3) as compared to the control group (M1) on day 3 (*p* = 0.043). At day 7, cell numbers in both groups increased and significantly outnumbered the control group (*p* = 0.027; Dunn's test, Figure 2(a)).

After 12 days, CFUs were observed in all three groups; however, the colony density in the inflammatory group and the anti-inflammatory group was significantly lower and demonstrated an altered cellular morphology (demonstrating more spindle-shaped morphology) compared to the control group (Figure 2(b)). CFUs' numbers in the inflammatory group (average of 43 colonies) and the anti-inflammatory group (average of 56 colonies) were strikingly lower than the control group (average of 360 colonies).

### 3.3. G-MSCs' Osteogenesis under Inflammatory/Anti-Inflammatory Environment

The osteogenic capability of G-MSCs under inflammatory and anti-inflammatory environment was evaluated by their osteogenic gene expressions on mRNA level and extracellular matrix calcification/nodule formation.

**Figure 3:**
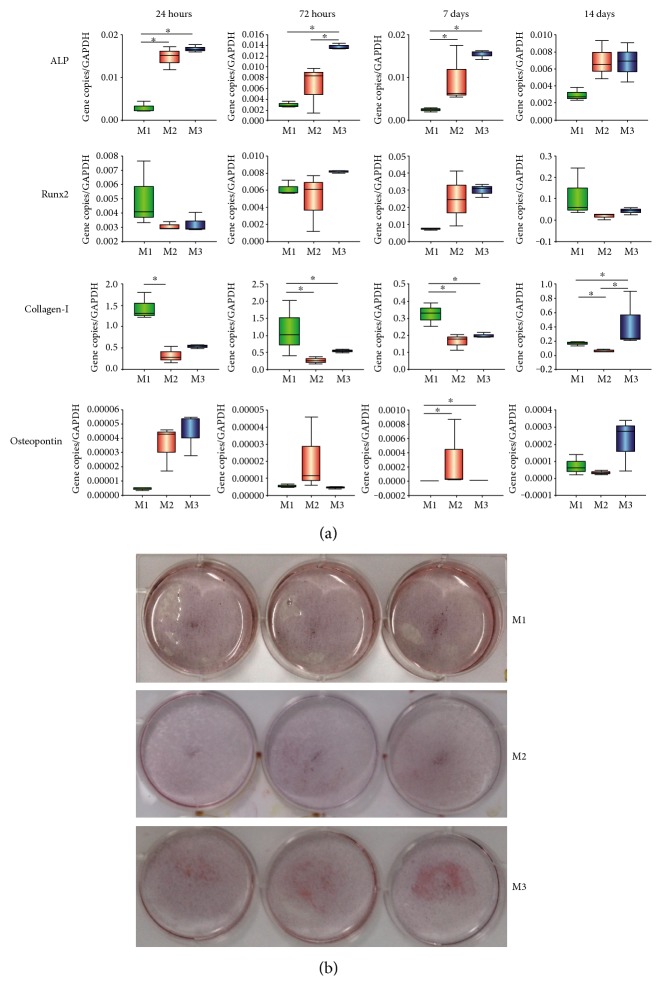
Osteogenic differentiation results of G-MSCs under inflammatory environment. (a) The mRNA expression of osteogenic genes (RUNX2, ALP, Col-I, and OPN) in the three groups were measured by real-time PCR at day 1, 3, 7, and 14 days. Expression level was normalized to GAPDH. Significant differences between groups are marked with an asterisk (^∗^*p* < 0.05; Dunn's test). (b) Alizarin red staining results of G-MSCs induced by osteogenic inductive medium (M1), inflammatory medium (M2), and anti-inflammatory treatment medium (M3) after 14 days. GAPDH: glyceraldehyde 3-phosphate dehydrogenase; Runx2: runt-related transcription factor 2; ALP: alkaline phosphatase.

RUNX2 showed higher expression levels in the anti-inflammatory group at day 1, day 7, and day 14 than those in the inflammatory group. ALP demonstrated higher expression levels in the anti-inflammatory group and the inflammatory group as compared to the control group until day 7 (*p* = 0.043). At day 3, ALP expression levels were significantly higher in the anti-inflammatory group than in the inflammatory group (*p* = 0.043). At day 14, ALP expression levels in the anti-inflammatory group and the inflammatory group were higher than in the control group (*p* = 0.066). The control group significantly expressed higher Col-I compared to the inflammatory group (*p* = 0.034 at day 1, *p* = 0.043 at day 3, *p* = 0.043 at day 7, and *p* = 0.043 at day 14) and the anti-inflammatory group (*p* = 0.043 at day 3 and day 7). At day 14, however, the anti-inflammatory group showed the highest Col-I expression, with significant differences observed compared to the inflammatory group and the control group (*p* = 0.043). OPN showed minimal expression level within all the tested groups. At day 7, OPN expression levels were significantly lower in the inflammatory group and the anti-inflammatory group, compared to the control group (*p* = 0.043). At day 14, the anti-inflammatory group showed the highest expression level among the three groups (*p* = 0.165; Dunn's test, Figure 3(a)).

After 14 days of osteogenic induction, cell cultures in all three groups were positively stained by Alizarin red. The number of calcified nodules in the anti-inflammatory group was higher than in the control group and in the inflammatory group (Figure 3(b)).

## 4. Discussion

G-MSCs, characterized by an easy isolation and a predictable *in vitro* expansion potential, are considered ideal, minimally-invasive alternative cellular sources of adult MSCs, with strikingly positive attributes for tissue engineering and regenerative approaches [10]. In the present study, G-MSCs showed all the stem/progenitor cell characteristics in accordance with the criteria defined by the International Society for Cellular Therapy [18].

Previous studies, testing the effect of single inflammatory cytokines on MSCs' behaviour reported biphasic and inconsistent results [19–23]. The combination of multiple inflammatory cytokines, forming a “cytokine cocktail” for cellular stimulation, mimics more closely the *in vitro* inflammatory environment, to which MSCs may be exposed during regenerative approaches [16, 24]. The “cytokine cocktail” (1 ng/ml IL-1*β*, 10 ng/ml TNF-*α,* and 100 ng/ml IFN-*γ*) used in the present study has previously been tested and proved to mimic the natural inflammatory environment, with an *in vitro* influences on cell proliferation, collagen synthesis, bone mineralization, and alkaline phosphatase activity of osteoblasts [25, 26] and embryonic stem cells (ESCs) [16]. In turn to selectively antagonize the effect of the proinflammatory “cytokine cocktail,” the present study used the two anti-inflammatory cytokines, IL-1ra and TNF-*α*i, in combination. Currently no antagonist to IFN-*γ* exists. Although this combination has been previously demonstrated to be beneficial in the treatment of various inflammatory diseases including rheumatoid diseases and periodontitis *in vivo* studies [6, 7, 27, 28], its effect on G-MSCs attributes has never been tested. Due to the difference in the affinity of IL-1 and its antagonist IL-1ra to their receptor [29], a tenfold IL-1ra concentration was employed for the competitive antagonism.

During their tissue regenerative approaches, G-MSCs, similar to all MSCs, pass through a primary cell division/proliferation phase followed by a subsequent differentiation stage to finally regenerate/repair the damaged tissues. Both cell numbers and differentiation capacity of MSCs are important to the final regenerative effect during clinical application [30]. In the present study, the inflammatory and anti-inflammatory crosstalk and their timed application significantly influenced G-MSCs' proliferation, colony-forming capability, and osteogenic differentiation potential.

At the primary stage of any stem/progenitor cells' mediated tissue regeneration approach, the fundamental requirement remains to be the presence of a large number of viable MSCs [31]. In contrast to previous studies, showing a deleterious effect of proinflammatory cytokines on cell proliferation of osteoblasts [16] and periodontal ligament stem cells (PDLSCs) [32] or almost no effect as on ESCs [16] and bone marrow mesenchymal stem cells (BMSCs) [33], similar to a recent investigation [13], inflammation enhanced the G-MSCs' proliferation, with an observed increase in cell numbers at 7 days. An anti-inflammatory treatment seemed unable to reverse this effect, as a significantly elevated cell numbers similar to the inflammatory group continued to be present at 7 days. Similarly, earlier investigations demonstrated no inflammation-mediated antiproliferative effect, with slightly increased MSCs' numbers after 7 days' culturing with TNF-*α*, IL-1*β*, or IL-1ra [33]. Aside from demonstrating clearly that G-MSCs could survive under inflammatory/anti-inflammatory challenge, the current results further proved that an initial short-term inflammatory stimulus, for up to 7 days, would be inductive for G-MSCs' proliferation and population expansion [13]. However, based on the reduced CFUs observed at 12 days, a continued inflammatory challenge beyond 7 days could significantly reduce the G-MSCs' proliferation/expansion potential.

Following the MSC proliferation/expansion phase, the cell differentiation stage begins to dominate the process of tissue regeneration [34]. In the current experiment, mRNA expression of genetic markers, representing early-to-later stages of osteogenesis (RUNX2, Col-I, ALP, and finally OPN), has been characterized, to investigate the osteogenic potential of G-MSCs under inflammatory/anti-inflammatory conditions [35–37]. Similar to previous studies [16, 24, 33], an inductive effect was not observed for the osteogenic capability of G-MSCs under inflammatory environment. This was evident through the suppression mRNA expression of most osteogenic markers including RUNX2, Col-I, and OPN and a compromised calcified deposit formation in the inflammatory group at 14 days. The observed negative effect on osteogenesis, especially at 14 days, could be attributed to the dose of the “cytokine cocktail” employed [33], as well as to the inflammatory stimulus duration. With the persistence treatment with the “cytokine cocktail,” a continued inflammatory environment could mediate intracellular signaling and production of NF-kappa *β* [13, 38], as well as a subsequent degradation of *β*-catenin [39], thereby interfering with the G-MSCs' osteogenic potential [16, 24].

Interestingly, and in clear contrast to previous investigations on BMSCs and osteoblasts [24, 32], inflammation upregulated the expression of ALP in G-MSCs, one of the earliest markers during MSCs' differentiation [40]. This was similar to previous reports [33], indicating that the activity of tissue-nonspecific alkaline phosphatase (TNAP) was significantly higher in human MSCs when cultured with proinflammatory cytokines IL-1*β*, TNF-*α*, and with their receptor antagonist, indicative of a possible ALP tissue-protective effect through dephosphorylation and detoxification process [41]. Hence similarly, the elevation of ALP mRNA expression under inflammatory/anti-inflammatory environment observed in the present study might indicate an active cellular reaction of G-MSCs to the multiple proinflammatory/anti-inflammatory cytokines present in their microenvironment.

On the other hand, in accordance with previous investigations [42–44], anti-inflammatory treatment reversed the inhibitory effect of inflammation on G-MSCs and boosted osteogenesis, as was evident by the elevated Alizarin red-positive deposits and the upregulation of all tested osteogenic markers observed at 14 days. The anti-inflammatory treatment appeared to strongly boost the cellular differentiation of G-MSCs by the inductive medium into an osteogenic lineage.

## 5. Conclusion

Combined, the current results qualify G-MSCs for tissue transplantation approaches in clinically challenging inflammatory environmental conditions, which normally represent the early phases of all physiological healing schemes. Furthermore, the results demonstrate for the first time how the cellular proliferation and osteogenic differentiation capacity of G-MSCs could be affected by inflammatory/anti-inflammatory synchronization. It seems that an initial short-term inflammatory phase would stimulate G-MSCs' proliferation. With the persistence of inflammation, this positive effect perished and was followed by a negative effect on osteogenesis. In this context, at the first stage of tissue regeneration, short-term controlled inflammation increases could enrich the cell numbers, a prerequisite for any tissue formation. While at the second stage, the subsequent subsidence of inflammation through an effective anti-inflammatory phase is essential for osteogenic cellular differentiation/maturation. However, further investigations are needed on the ideal modes and time points of inflammation/anti-inflammation agent-controlled delivery to affect cellular attributes. This timed scheme of an initial inflammatory stimulation, followed by a subsequent anti-inflammatory treatment, could hold a remarkable potential in the field of G-MSC-mediated osteogenic tissue regeneration.

## Figures and Tables

**Table 1:** Primer names and ID used for real-time PCR (supplied by Roche).

Gene	Assay ID	Gene symbol	Accession ID
GAPDH	141,139	H. sapiens 100,071,074	NM_002046
RunX2	113,380	H. sapiens 100,109,195	ENST00000359524
ALP	103,448	H. sapiens 100,109,186	ENST00000374840
Col-I	100,861	H. sapiens 100,109,140	ENST00000225964
OPN/SPP1	101,170	H. sapiens 100,109,168	ENST00000395080

RT-PCR: reverse transcription-polymerase chain reaction; GAPDH: glyceraldehyde 3-phosphate dehydrogenase; Runx2: runt-related transcription factor 2; ALP: alkaline phosphatase; Col-I: type I collagen; OPN: osteopontin.
